# Immunophenotype Rearrangement in Response to Tumor Excision May Be Related to the Risk of Biochemical Recurrence in Prostate Cancer Patients

**DOI:** 10.3390/jcm10163709

**Published:** 2021-08-20

**Authors:** Paulius Bosas, Gintaras Zaleskis, Daiva Dabkevičiene, Neringa Dobrovolskiene, Agata Mlynska, Renatas Tikuišis, Albertas Ulys, Vita Pašukoniene, Sonata Jarmalaitė, Feliksas Jankevičius

**Affiliations:** 1 Faculty of Medicine, Institute of Clinical Medicine, Vilnius University, 03101 Vilnius, Lithuania; paulius.bosas@nvi.lt (P.B.); feliksas.jankevicius@santa.lt (F.J.); 2 National Cancer Institute of Lithuania, 08406 Vilnius, Lithuania; daiva.dabkeviciene@nvi.lt (D.D.); neringa.dobrovolskiene@nvi.lt (N.D.); agata.mlynska@nvi.lt (A.M.); renatas.tikuisis@nvi.lt (R.T.); albertas.ulys@nvi.lt (A.U.); vita.pasukoniene@nvi.lt (V.P.); sonata.jarmalaite@nvi.lt (S.J.); 3 Department of Chemistry and Bioengineering, Vilnius Gediminas Technical University, 10223 Vilnius, Lithuania; 4 Life Sciences Center, Institute of Biosciences, Vilnius University, 10257 Vilnius, Lithuania; 5 Vilnius University Hospital Santaros Klinikos, 08661 Vilnius, Lithuania

**Keywords:** prostate cancer, prostatectomy, biochemical recurrence, immunophenotyping

## Abstract

Background: Prostate cancer (PCa) is known to exhibit a wide spectrum of aggressiveness and relatively high immunogenicity. The aim of this study was to examine the effect of tumor excision on immunophenotype rearrangements in peripheral blood and to elucidate if it is associated with biochemical recurrence (BCR) in high risk (HR) and low risk (LR) patients. Methods: Radical prostatectomy (RP) was performed on 108 PCa stage pT2–pT3 patients. Preoperative vs. postoperative (one and three months) immunophenotype profile (T- and B-cell subsets, MDSC, NK, and T reg populations) was compared in peripheral blood of LR and HR groups. Results: The BCR-free survival difference was significant between the HR and LR groups. Postoperative PSA decay rate, defined as ePSA, was significantly slower in the HR group and predicted BCR at cut-off level ePSA = −2.0% d^−1^ (AUC = 0.85 (95% CI, 0.78–0.90). Three months following tumor excision, the LR group exhibited a recovery of natural killer CD3 − CD16+ CD56+ cells, from 232 cells/µL to 317 cells/µL (*p* < 0.05), which was not detectable in the HR group. Prostatectomy also resulted in an increased CD8+ population in the LR group, mostly due to CD8+ CD69+ compartment (from 186 cells/µL before surgery to 196 cells/µL three months after, *p* < 001). The CD8+ CD69+ subset increase without total T cell increase was present in the HR group (*p* < 0.001). Tumor excision resulted in a myeloid-derived suppressor cell (MDSC) number increase from 12.4 cells/µL to 16.2 cells/µL in the HR group, and no change was detectable in LR patients (*p* = 0.12). An immune signature of postoperative recovery was more likely to occur in patients undergoing laparoscopic radical prostatectomy (LRP). Open RP (ORP) was associated with increased MDSC numbers (*p* = 0.002), whereas LRP was characterized by an immunity sparing profile, with no change in MDSC subset (*p* = 0.16). Conclusion: Tumor excision in prostate cancer patients results in two distinct patterns of immunophenotype rearrangement. The low-risk group is highly responsive, revealing postoperative restoration of T cells, NK cells, and CD8+ CD69+ numbers and the absence of suppressor MDSC increase. The high-risk group presented a limited response, accompanied by a suppressor MDSC increase and CD8+ CD69+ increase. The laparoscopic approach, unlike ORP, did not result in an MDSC increase in the postoperative period.

## 1. Introduction

Prostate cancer (PCa) is the second most common cancer diagnosis made in men and the fifth leading cause of death worldwide [1]. PCa is also known to exhibit a wide spectrum of aggressiveness, which is sometimes not easy to classify during the early stage of diagnosis. Recurrence after radical prostatectomy (RP) is not uncommon for patients with PCa, and it is highly dependent on various risk factors [2,3,4].

PCa involves a relatively slow-growing tumor, but it is also known to be one of the few malignancies capable of delayed recurrence [2]. The postoperative course of PCa results in biochemical recurrence (BCR), even in the low-risk (LR) group, beginning at roughly a four-year time point following RP, in ~25% of patients [3]. The cumulative risk of 10-year incidence of BCR in an intermediate-risk group is up to 32% [5]. Men with high-risk (HR) PCa present a serious clinical challenge, where surgery alone is rarely performed and intervention must be considered in the context of multimodality treatment. The proportion of PCa cases classified as HR recently increased worldwide (for example, reaching more than 20% of all PCa cases in the U.S.), and the use of RP in this population rose significantly as well [6].

Regardless of the risk category, tumor excision, per se, exhibits adverse systemic effects known to influence PCa treatment outcomes significantly. The surgical procedure itself leads to an increase of tumor cell release into the circulation and upregulation of adhesion molecules in target organs, resulting in facilitated metastatic spread after RP. Surgery also suppresses anti-tumor immunity, allowing circulating cells to survive [7]. The mechanisms behind activation of this surgery-induced immunological dysfunction are poorly understood. Certain evidence indicates the “concomitant immunity” accompanying primary tumor growth [8]. Concomitant immunity is a unique phenomenon that elicits an immune response insufficient to destroy the primary tumor but able to prevent a secondary tumor from growing. Therefore, the excision of a primary tumor might turn into a signal-provoking proliferation of micrometastases, which were dormant before surgical intervention. In fact, more than 70% of PCa patients are known to have preoperative bone marrow dissemination, regardless of stage, Gleason score, PSA, or any evidence of systemic disease [9]. The impact of surgery-provoked progression or surgery-induced immunosurveillance profile on this early micrometastatic disease are not known. Surgical excision also eliminates all immune cells that were fixed inside the tumorous tissue. Remarkably, these tumor-infiltrating immune cells are known to reciprocally influence systemic immunosurveillance and metastatic behavior in the preoperative period [8], meaning that tumor removal should disrupt the systemic and local immunity communication. The question arises whether this disruption might be reflected in phenotypic alteration of peripheral blood lymphocytes. It has long been known that surgery itself has immune consequences in terms of temporary depleted numbers of T lymphocytes, B lymphocytes, natural killer (NK) cells, and HLA-DR monocytes [10,11]. Laparoscopic surgery appears to spare the immune system significantly more in these cases. Additionally, there is a growing body of evidence to suggest that attenuation of the surgical inflammatory stress may reduce postoperative tumor recurrence or metastatic dissemination [12].

The oncological outcomes and complication rates from LRP and ORP are similar [13]. However, the comparison of immunological rearrangements between these two methods has not been investigated in PCa. PCa is a unique tumor, providing well-established criteria for postoperative follow-up by PSA. Unlike other tumor markers, PSA is organ specific and undergoes rapid postoperative decline, with a well-detectable plateau during the one- to three-month period. We selected this timeframe to examine immunophenotype correction induced by surgery to avoid early stress-induced hormone and cytokine ejection.

Many cancers, including PCa, are known to elicit an overproduction of a range of immunocyte suppressors, including immature myeloid cells, which recently were categorized as myeloid-derived suppressor cells (MDSCs) [14,15,16,17]. Tumor excision response of MDSC in PCa patients has also been reported [15]. The mechanisms of this MDSC response are closely related to a disruption of tumor and monocyte interaction. Monocytes from the blood of prostate cancer patients can fully mature to dendritic cells only after the PCa excision is accomplished [15]. In addition, the direct link between MDSC increase and presence of the primary tumor in the abovementioned study was a specific and distinguishing immunosuppressive indicator of PCa but not colorectal cancer. Deeper insight into MDSC response to RP with regard to a risk group is needed.

In this study we hypothesized that there must be a new balance established postoperatively between systemic immunity and the residual tumor. A distinct signature of rearrangements was attributed to the LR (postoperative increase in CD8+, CD8+ CD69+, CD16+ CD56+, and stable myeloid-derived suppressor cell (MDSC)) versus HR (postoperative increase in CD8+ CD69+ and suppressor MDSC increase) group. ORP, but not LRP, was more likely to result in postoperative increase of suppressor MDSCs.

## 2. Materials and Methods

### 2.1. Patients and Surgical Procedure

This study was approved by the National Review Board (Vilnius, Lithuania, 158200-17-928-442), and written informed consent was obtained from each study participant. All methods were performed in accordance with the relevant Lithuanian national guidelines and regulations. In total, 108 patients with PCa were enrolled. The eligibility criteria for enrollment were as follows: (1) no history of diagnosis or treatment for other malignancies, (2) no androgen deprivation therapy (ADT) or radiotherapy (RT) before surgery and 3 months postoperatively, (3) no inflammatory condition, immunosuppressive intervention, or presence of autoimmune diseases, (4) no perioperative blood transfusions, (5) preoperative and postoperative (up to 3 months) white blood cell (WBC) count less than 10,000 µL^−1^, and (6) liver enzymes, glomerular filtration rate, C-reactive protein, and bilirubin in the normal range. The clinical stage was assessed according to the 2002 TNM staging guide, prostate biopsy cores were obtained using a >10-core biopsy protocol, and preoperative PSA was measured before digital rectal examination. The biopsy and pathologic gradings were assessed according ISUP Gleason score. The pT stage was graded according to the 2002 AJCC staging system for PCa. The clinicopathological data of study participants are listed in Table 1.

The LRP extraperitoneal prostatectomy was performed using a five-trocar technique for 63 (58.3%) patients. The prostato-vesical junction was incised and the vas deferens and seminal vesicles were dissected. The prostate was dissected in an antegrade fashion. The urethra was transected following separation of the dorsal venous complex. A running suture vesicourethral anastomosis was placed. Conventional open radical retropubic prostatectomy was performed for 45 (41.7%) patients in a retrograde fashion extraperitoneally following dissection of the urethra. The urethro-vesical anastomosis sutures used an interrupted stitch. Propofol total intravenous anesthesia without sevoflurane or opioid was applied to all study participants. Blood samples were collected before and at 1- and 3-month time points after RP. Patient follow-up included PSA measurements every 3 months for two years and then every 6 months after that. Biochemical recurrence (BCR) was defined as a PSA value of 0.2 ng/mL after RP, confirmed by at least two consecutive measurements. BCR-free survival was calculated from the date of the surgery to the date of the diagnosis of the BCR. Patients were separated into two subgroups according to their risk of progression. The high-risk (HR) group was defined as having at least one of the following criteria: Gleason score ≥ 4 + 3, lymph node involvement (N1), pathological stage ≥ pT3a, and positive surgical margins. All other participants were attributed to a low-risk (LR) category.

### 2.2. Flow Cytometry and PSA Analysis

Blood was obtained by venipuncture and collected in BD Vacutainer^®^ tubes containing EDTA anticoagulant (BD Biosciences, San Jose, CA, USA). Tubes were then rotated on a shaker until the phenotypic staining procedure and flow cytometry analysis were performed (30 min–6 h post collection).

For peripheral blood analysis, 100 μL of blood was added to the four appropriate tubes, and cells were processed according to the manufacturer’s instructions. A total of four tubes per patient were used and stained with the following antibodies: tube (1): anti-CD56-PE/anti-CD16-APC/anti-CD3-FITC/anti-CD19-BV421^TM^/anti-CD45-PerCP (BioLegend, San Diego, CA, USA); tube (2): anti-CD25-PE/anti-CD4-FITC/anti-CD3-APC; anti-FoxP3-BV421^TM^ (BioLegend, San Diego, CA, USA); tube (3): anti-CD8a-FITC/anti-CD69-APC/anti-CD3-BV510^TM^ (BioLegend, San Diego, CA, USA); tube (4): anti-HLA-DR-PE/anti-CD14-FITC/anti-CD11b-BV421^TM^/anti-CD33-APC (BioLegend, San Diego, CA, USA). The NK cell was defined as CD3 − CD16+ CD56+, total MDSCs were defined as CD45 + CD3 − CD19 − CD56 − CD16 − HLA-DR − CD33 + CD11b+, and the T reg cell definition was CD4+ CD25+ FoxP3+. A total of 20 µL of each antibody was added to the appropriate tube. The blood was incubated with the antibodies for 15 min in darkness, followed by red blood cell lysis with BD FACS Lysing solution (BD Biosciences, San Jose, CA, USA) for 15 min in darkness. Cells were then washed twice in BD-Cell-Wash solution (BD Biosciences, San Jose, CA, USA) and fixed in BD-Cell-Fix solution (BD Biosciences, San Jose, CA, USA) prior to data acquisition. All processed samples were then analyzed on a BD LSR II System flow cytometer (BD Biosciences, San Jose, CA, USA). A total of 20,000 events were acquired, and BD FACSDiva™ Software (BD Biosciences, San Jose, CA, USA) was used for subset analysis. The immunophenotyping, complete blood cell count (CBC), and PSA analysis were performed from the blood samples collected at the same time points during venipuncture. The CBC blood analysis was conducted using a Sysmex NX-1000 (Sysmex Europe, Norderstedt, Germany). The PSA data from the serum were obtained exploring an ultrasensitive PSA assay (sensitivity limit 0.002 ng/mL, Cobas e411, Roche Diagnostics, Risch-Rotkreuz, Switzerland).

ePSA was calculated as follows:

(1) $$\mathrm{ePSA}=\frac{100}{\mathrm{HT}}=\frac{100\times \ln\left(\frac{N}{M}\right)}{\ln2\times \left(∆t\right)}$$

where HT = half-life time of serum PSA in days, N = current PSA, and M = previous PSA. ePSA values < 0 appear if PSA is decreasing and > 0 if increasing. Δt = time interval, expressed in days between the two PSA measurements. The ePSA metric is “percent/day”, reflecting growth or elimination fraction.

PSA value adjustment to a specific sampling day (e.g., day 30) was done using the following equation:

(2) $${\mathrm{PSA}}_{\mathrm{day}30}={\mathrm{PSA}}_{M}\times 2^{\left(M-30\right)/\mathrm{HT}}$$

where PSA_day30_ = PSA value as if it was measured on day 30, PSAM = actual PSA value at M representing a “one month” time point, M = number of days from RP to PSA measurement, and HT = half-life time of PSA from RP to M time point. PSA_day91_ was used to adjust values to represent a “three month” time point.

### 2.3. Statistical Analysis

IBM-SPSS Statistics 21 (SPSS, Inc., Chicago, IL, USA) was used for data analysis. Data were summarized by frequency and percentage for categorical variables and by median and range for continuous variables. All statistical tests were two sided, and differences were considered statistically significant when *p* < 0.05.

## 3. Results

**Figure 1:**
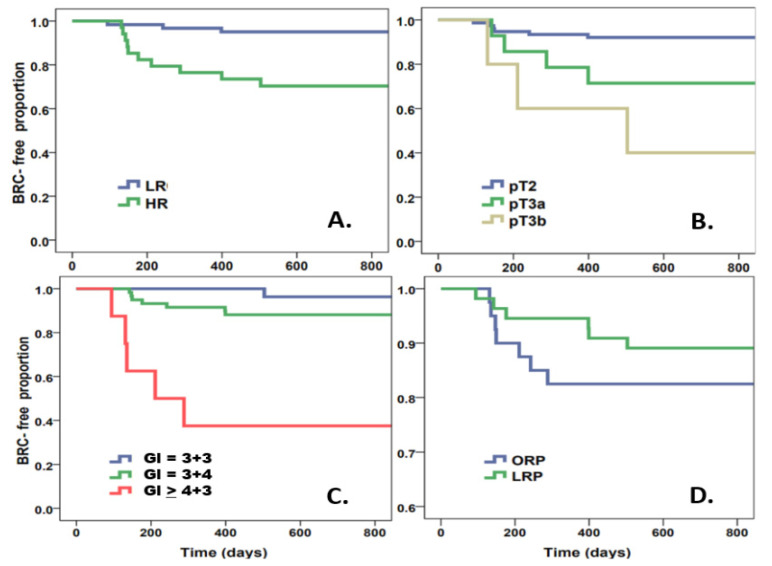
Kaplan-Meier survival curve of biochemical recurrence-free survival of postoperative patients according to risk (low risk versus high risk) (**A**); pathological status (**B**); Gleason score (**C**); surgical method: LRP vs. ORL (**D**). Stratification by surgical method showed no significant difference between methods applied (log rank p = 0.36).

During the median follow-up time of 32 months, BCR occurred in 12 out of 83 patients (14.5%) (patients with postoperative early ADT or RT were excluded). There was a significant difference in proportion of BCR-free survival between patients with HR or LR (Figure 1A, log rank *p* < 0.001); with pT2 vs. pT3 (Figure 1B, log rank *p* < 0.001) and Gleason score (Figure 1C, log rank test *p* < 0.001). There was no significant difference between the BCR-free survival rates of patients undergoing ORP or LRP (Figure 1D, log rank *p* = 0.33).

**Figure 2:**
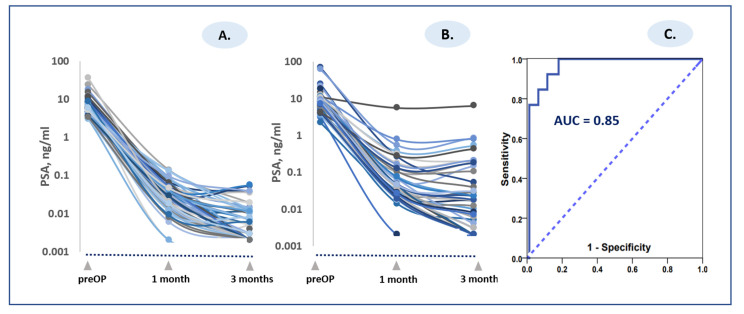
Individualized PSA response to radical tumor excision. (**A**) Low-risk group, (**B**) high-risk group, (**C**) ROC curve for predicting BCR. Abbreviations: PreOp: values before RP; one month and three months: PSA values at one-month and three-month time point postoperatively.

Tumor excision resulted in a significant PSA decline. Preoperative and postoperative PSA values in the LR and HR patient groups are shown in Table 2. Postoperative PSA in 25 (23.1%) patients at the one-month time point was less than 0.02 ng/mL, whereas 81 (75%) patients remained at a PSA level above or equal to 0.02 ng/mL. At the three-month time point, 61 (56.5%) patients exhibited PSA values below 0.01 ng/mL and 43 (39.8%) patients above or equal to 0.01 ng/mL. A PSA value below 0.01 ng/mL during the two- to three-month postoperative period is indicative of favorable surgical treatment outcomes. In addition to absolute PSA evaluation, we attempted to explore the kinetic PSA decay parameter and to compare it to BCR occurrence. The rate of PSA decline was estimated by half-life time (HT) of PSA decay (Table 2). The PSA decline rate was similar between HR and LR patients at the one-month postoperative time point. Comparing PSA rates between HR and LR patients in the interval between one month and three months postoperatively revealed significant differences. The PSA values continued to decline in the time interval between one month and three months in the LR group, whereas a significant slowdown or rebound of PSA values was observed in the HR group (negative HT values in Table 2 are indicative of PSA rebound). The HT value is known to hold a mathematical drawback when the curve is flattening or changing direction (PSA decline vs. PSA increase for the same patient). Therefore, we used ePSA values [18] to further evaluate PSA dynamics and their relationship to BCR in the two risk groups. The ePSA index specifies a percentage of PSA change per day, with positive values indicating PSA rise and negative values indicating PSA decline. The calculated ePSA value at the interval between one and three months was −2.8 + 1.54% d^−1^ (range from −0.35% d^−1^ to +0.35 % d^−1^, median −3.19% d^−1^) for the LR group versus +2.9 + 0.30% d^−1^ (range from −0.17% d^−1^ to +0.06 % d^−1^, median −3.48% d^−1^) for the HR group. Thus, in the one- to three-month interval, the tendency of PSA rebound, or at least the slowdown of decline, was more pronounced in the HR patient group (Figure 2A,B; Table 2). The cut-off level of ePSA > −2.0% d^−1^ for predicting BCR was AUC = 0.96 (95% CI, 0.919–1.00) (Figure 1C).

**Figure 3:**
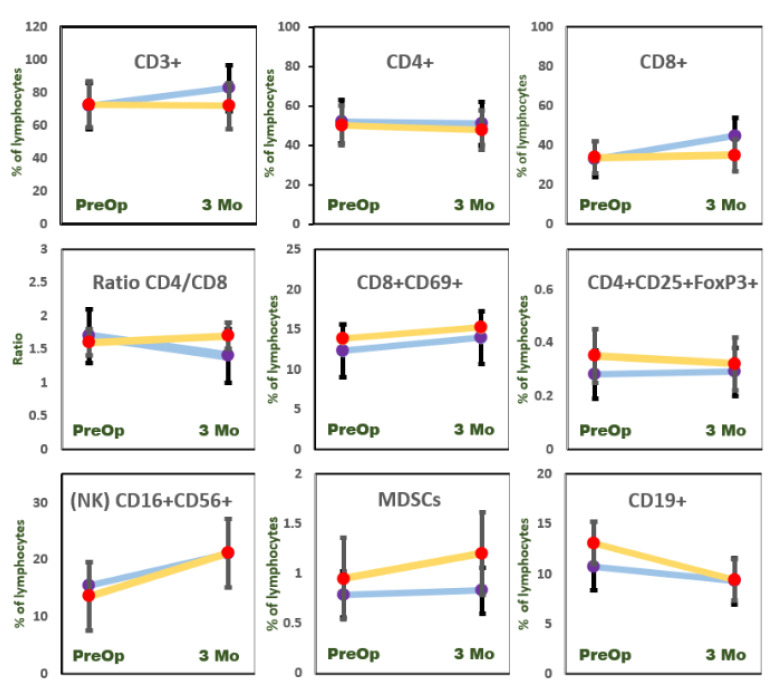
Prostatectomy-induced percentage change of immunocyte populations. Low-risk (blue line) and high-risk (yellow line) patients were tested before surgical intervention (PreOp) and three months after (3 Mo). Bars represent standard error of mean value. All values are calculated as a percent of total lymphocytes, with the exception of CD4/CD8 ratio.

The change in immunocyte phenotype was rather modest as compared to PSA decline (Figure 3). The effect of RP on the immunophenotype rearrangement is shown in Table 3 and Figure 3. Tumor excision resulted in a T cell (CD3+) increase (*p* < 0.001) in the LR group, but no effect was seen in the HR group three months after RP (*p* = 0.11). This T cell increase was associated with CD8+ but not CD4+ compartment, resulting in a significant postoperative CD4/CD8 ratio decrease in the LR group. None of the total CD4+ or CD8+ postoperative rearrangements were observed in HR patients. The cytotoxic T cell subset CD8+ CD69+ saw a surgery-mediated increase in both the LR (*p* < 0.001) and HR (*p* < 0.001) groups.

Tumor removal exhibited no effects on Treg compartment (CD4+ CD25+ FoxP3+) in either group. The NK cell (CD3 − CD16+ CD56+) significantly recovered after RP in LR (*p* < 0.05) patients but not in HR (*p* = 0.24) study participants. The suppressor MDSCs increased postoperatively only in the HR group (*p* < 0.05).

Comparing the ORP vs. LRP technique revealed differences in the immunocyte restorative capacity of the two surgical methods. The difference was observed for suppressor MDSCs, which were significantly increased following ORP but not LRP (Table 4). The postoperative CD3-CD16+ CD56+ increase was observed in both groups: LRP and ORP. Postoperative CD3+ and CD8+ subset increase was a characteristic feature of LRP surgical technique but not ORP.

## 4. Discussion

Tumor excision was accompanied by a dramatic decline in PSA and rather modest rearrangements of peripheral immune cells assessable at one and three months postoperatively. The rate of postoperative PSA decline (ePSA) did vary greatly as compared to absolute PSA values (CV numbers of ePSA were roughly 10 times lower as compared to PSA values; data not shown). This indicates that the surgical excision resulted in the elimination of a significant proportion of prostate tissue and adjacent infiltrated tissues capable of producing PSA. Elimination of PSA from blood also meant that chronic exposure of tumor-associated antigens to immunocyte was significantly reduced. We studied the rearrangements of the immune system at one and three months in order to avoid early stimulus of postoperative stress (stress hormone ejection, WBC, and cytokine fluctuations). The high immunogenicity of PCa [19] offers a unique platform to study tumor excision effects on sparing or even boosting postoperative immunosurveillance function. However, the specific markers indicating immune rearrangement in progressing PCa or following RP are yet to be elucidated [20]. We compared pre- and postoperative status in patients with primary PCa, subdivided into two arbitrary groups: LR (including low- and intermediate-risk patients) and HR (including high- and very high–risk patients). RP induced a CD8+ percent and absolute count increase in LR patients, which was not the case in the HR group. Tumor resection is known to release antitumor CD8^+^ T cells from chronic antigen exposure, allowing a gradual differentiation toward functional antitumor memory T cells [21]. The absence of T-cell CD8+ recovery in our HR group might indicate the continuation of postoperative chronic antigen exposure. The indirect evidence for this is demonstrated by substantially slower PSA decline rates in HR patients, reflected in their ePSA. The mean value of ePSA in HR patients turned positive in the interval between one and three months, which indicates a partial rebound of PSA in this group. Although PSA cannot be directly associated with tumor-associated antigens, its partial rebound might signify prolonged tumor antigen or another tumor released product exposure. Surgical stress might be an additive factor disabling cytolytic CD8+ effectiveness during this chronic exposure phase. In fact, surgically stressed CD8+ T cells display reduced cytokine secretion, proliferation, and tumor infiltration in response to tumor antigens [22].

We did not observe any significant change in CD4+ numbers following RP in either risk group. This stable CD4+ level resulted in surgery-induced CD4/CD8 ratio decline in the LR group, due to CD8+ subset increase. Some investigators tend to attribute this effect to early postoperative stress. Thus, modest stress was found to decrease the ratio of CD4/CD8 in the spleen of mice [23]. Other studies reported that postoperative correction of CD4/CD8 ratio and improvement of prognosis might be achieved by adding adoptive immunotherapy [24]. The number of intratumoral rather than circulating CD8+ cells is considered indicative of immunosurveillance by some authors. Thus, the higher CD8+ counts in PCa tissue were associated with a lower risk of BCR and metastatic disease [25]. However, the opposite effect with an increased CD8+ intratumoral density was also reported [26].

Memory CD8+ T cells are classified as those that are present in circulation and those that are tissue-resident or tumor-resident memory CD8+ T cells. Surgical intervention disrupts this communication. Therefore, it is hard to interpret the recovery of circulating CD8+ T cells, which was observed by us only in the LR group, not in HR group. We hypothesized that chronic exposure to tumor or residual PCa products remaining postoperatively were inhibiting circulatory CD8+ subset in the HR group. The number of T cells co-expressing CD69+ and CD8+ increased in both risk groups postoperatively. The absolute numbers of CD8+, however, were not increased in HR patients. It was demonstrated that rapid recruitment of memory CD8+ from the circulation into non-lymphoid tissues was accompanied by the co-expression of CD69+ and subsequent residence in the infiltrated sites [27]. This observation was made on non-malignant but virus-infected tissues. However, the mechanisms involved in the memory CD8+ T cells acquiring CD69+ upon de novo antigen stimulation should be similar.

We can hypothesize that higher CD8+ counts remaining in circulation postoperatively might be also preconditioned to elicit increased acquisition of CD69+. This might lead to a recruitment of CD8+ CD69+ T cells into residual or micrometastatic sites. The CD8+ CD69+ cell activation has been shown to be a key factor in tumor control, at least in experimental models [28]. Interestingly, the most appealing evidence indicating that the circulatory CD8+ CD69+ cells are a key factor in antigen-related tissue destruction was demonstrated by histological findings of a heart transplant rejection specimen [29].

PCa destruction induced by radiotherapy RP revealed an opposite immunophenotype rearrangement profile compared to ours [30]. In that study, the CD4/CD8 ratios in peripheral blood were consistently higher in patients with a complete response or partial response to RT than in those classified to have stable disease. A direct comparison between tumor excision and RT-mediated tumor destruction might not be relevant since RT can also result in a severe treatment-related lymphopenia.

We also expected that CD4+ CD25+ Fox3P+ regulatory T cells, which include two categories of lymphocytes (natural and induced Tregs) will be responsive to PCa removal. In fact, prostate infiltrating CD4+ Fox3P+ cells were shown to elicit pro-tumorigenic activity even before malignant transformation was established [31]. The T regs and MDSCs are currently viewed as one of the key targets to achieve therapeutic effects in PCa patients treated with immune checkpoint inhibitors (ICIs) [32]. However, we did not observe any PCa excision effects on the T reg CD4+ CD25+ Fox3P+ cells among study participants. Calculating ratios of CD4/CD4+ CD25+ FoxP3+ did not add any additional information.

We observed a postoperative MDSC increase in the HR group only. It indicates that the immune restoration effect of surgical treatment is harder to achieve in HR patients. An important finding was that the RP made by the minimally invasive technique, LRP, did not result in an increase of MDSCs among PCa patients. The immunity-sparing LRP, as compared to ORP, is potentially advantageous in terms of not upregulating MDSCs capable of provoking dormant tumors. An increasing body of evidence demonstrates that immunosuppressive mechanisms mediated by MDSCs are a key contributor to tumor progression and that these mechanisms promote tumor escape from dormancy [16]. The potential LRP benefit of preventing surgery-induced MDSC upregulation did not result in better treatment outcomes in our study participants. The frequency of BCR was comparable in LRP vs. ORP groups, with a trend of LRP advantage (*p* = 0.36). However, mechanisms of MDSC-mediated immune evasion of dormant tumors might take many years to be triggered.

We observed an NK number increase in the LR group only. A similar NK response to PCa surgery was recently found by Lu et al. [33]; only functional NK restoration following RP was observed. In addition, the absence of positive margins facilitated surgery-induced NK restoration significantly in this study. Our risk group assessment was slightly different from the one explored in [33]. We did not investigate functional NK activity, but NK counts were increased in the LR group following tumor excision. The restorative effect of RP on NK activity might be expected. Advanced cancer stages are known to elicit NK activity suppression significantly more as compared to initial stages [34]. Therefore, different response patterns in LR and HR patients can be explained by the size and extension of the operated tumors.

Both the LRP and ORP restored NK counts significantly among our study participants. A significantly lower impairment of cell-mediated immunity after surgery was demonstrated in patients treated by the laparoscopic method compared with those treated by open laparotomy [9,10]. However, other studies failed to detect any difference in immunophenotype rearrangements when these two methods were compared [11]. All of these findings, however, were obtained from non-PCa patients. This is due to the fact that the LRP technique is not yet universally considered to be the golden standard for PCa surgical treatment. However, LRP for the excision of PCa is being increasingly adopted as the method of choice [35,36,37]. National US cohort data showed that LRP was independently associated with clinically meaningful reductions in positive surgical margins, postoperative radiation therapy, and 30-day mortality, compared to the open method [38]. There is, however, a certain amount of skepticism [39] among some urologists about the advantages of this method. Nevertheless, accumulating evidence suggests that the LRP technique not only results in less pain and faster recovery, but also a specific biomedical advantage during the postoperative course of PCa. For instance, there are data indicating that the LRP technique could be advantageous in terms of a lower release of circulating tumor cells [40] or fewer blood transfusions needed in PCa patients [41]. These two factors might be essential for immunological crosstalk between immunocytes and residual micrometastatic disease. The association between higher risk of recurrence and transfusion rates [42] or circulatory tumor cell number [43] is well documented. The auto- or alloantigen overstimulation in these two cases might also play a negative role.

Overall, different postoperative immunocyte profiles of LRP (CD8+ ↑, NK ↑, MDSC≈) vs. ORP (CD8+ ≈, NK ↑, MDSC ↑) were observed. The ORP-induced MDSCs in some patients might lead to a chain of events in the postoperative period. The importance of MDSC increase in postoperative period has been reported in lung cancer patients [43]. This study demonstrated that MDSCs increased after lung cancer surgery, and surgery-induced MDSCs correlated significantly with elevated numbers of Treg in circulation. Experimental surgery-induced lung tumors were related to MDSC increase in this study. We did not observe any significant increase in Treg, regardless of the risk group or surgical method applied. The postoperative increase in circulating MDSCs observed in our ORP group and in HR patients might also lead to some adverse effects, such as angiogenesis activation. For instance, the MDSCs isolated after surgery from lung cancer patients were more efficient in promoting angiogenesis and tumor growth than MDSCs isolated before surgical operation [44]. A potentially less invasive approach not upregulating suppressor MDSC release in the postoperative period might be advantageous over ORP.

The LRP adoption for RP might have important implications, since a minimally invasive technique is known to elicit a distinct range of systemic effects. For instance, a reduction of surgical trauma by use of a laparoscopic approach was demonstrated to restore reduced IL-2, TNF, and INF production by T cells [45]. This effect was not seen where open surgery was applied. The experimental tumor progression was significantly reduced by the laparoscopic approach, but not by laparotomy [46]. In addition, immune enhancement in some models was seen by exploring a minimally invasive technique [47]. Finally, LRP does not cause significant increases in circulating PCa tumor cells, in contrast to ORP [40].

PCa exhibits a paradoxical interplay with the immune system. Although highly immunogenic, PCa is rarely responsive to immunomodulating therapies, and the PD-L1 expression in PCa cells is generally low [48].

It is well known that the systemic effects following surgical intervention might result in a series of events that could significantly provoke the progression of a malignant disease [7,49]. However, the opposite concept also exists: surgical tumor removal restores blocked antitumor immunity [50] and paves the way for perioperative immunotherapy. The surprising evidence in support of the latter concept comes from an analysis of debulking surgeries applied to oligometastatic PCa [51]. This controversial approach to conduct a surgical procedure on metastatic PCa seems to be justifiable in some cases [52]. The evidence in support of this concept is based on experiments with mice becoming immune to tumor rechallenge following the excision of a primary tumor [50].

In our study, HR and LR patients demonstrated distinct subset responses to RP. The patterns for the LR group can be described as restorative (T-cell↑, CD8+ CD69+↑, NK↑, MDSC≈), whereas the HR group can be characterized as insufficiently restorative (CD8+ CD69+↑, MDSC↑) and potentially unable to control minimal residual disease.

Our study is not without certain limitations. First, the study period was not long enough to track all BCR patients. Second, we explored arbitrary risk groups, combining low and intermediate risk in the LR category and high and very high into the HR category. This categorization enabled us to demonstrate two distinct patterns of immunocyte behavior in response to surgical intervention. Surgery-induced immunocyte recovery might not always mean therapeutic gains. For instance, tumor excision was shown to elicit dramatic down-regulation of the peripheral CD279 (PD-1) subset [53]. But at the same token, it meant that patients with surgery-reconstituted immunity became unresponsive to ICI. Therefore, the authors suggest that the opportunity for ICI therapy only exists in the timeframe of the early perioperative period, while the CD279 cell is still present. Our study addresses the issue that surgery-induced immunosurveillance recovery is quite complex, and multiple markers beyond CD279 might be playing a role. Clinical trials applying perioperative immunotherapies for PCa should consider specific profiles of immunocyte subsets emerging after tumor excision.

Our data exhibits direct clinical implications in cases where patients might need postoperative RT. The appropriate timing of RT for HR patients after RP remains controversial. The efficacy of adjuvant RT vs. salvage RT seems to be advantageous [54], although some clinicians might show resistance to acknowledge the necessity of timely RT [55]. Our data show that not only HR patients but also some participants in the LR group might be at higher odds of developing BCR, as judged by ePSA and some immunophenotype alterations. Unlike immunological analysis, the ePSA is easy to implement in clinical practice. It requires only two PSA tests at the early postoperative period, with an exact timing expressed in days. At least in theory, more patients might benefit significantly from timely adjuvant RT, since ePSA is an indicative parameter in all risk groups.

We conclude that tumor excision in prostate cancer patients results in two distinct patterns of immunophenotype rearrangement. The low-risk group was highly responsive, revealing postoperative restoration of T cells, NK cells, and CD8+ CD69+ and the absence of suppressor MDSC increase. The high-risk group presented a surgery limited response status accompanied by a suppressor MDSC increase and CD8+CD69+ increase. The laparoscopic approach, unlike ORP, did not result in an MDSC increase in the postoperative period.

## Figures and Tables

**Table 1:** Clinicopathological characteristics of patients.

Patient Characteristics	
No of patients (%)	108 (100%)
Age (years)	
<61	40 (37.0%)
61–65	29 (26.9%)
>65	39 (36.1%)
Preoperative PSA (ng/mL)	
<4	15 (13.9%)
4–10	71 (65.7%)
>10	22 (20.4%)
White blood cells (k/µL)	6.02 (5.1–8.0)
Lymphocytes (k/µL)	2.12 (1.6–2.7)
Tumor characteristics	
Extracapsular extension	28 (25.9%)
Seminal vesical invasion	12 (15.7%)
Lymph node involvement	5 (4.6%)
pT stage	
pT2	78 (72.2%)
pT3	30 (27.8%)
Gleason score	
Grade 1 [≤3 + 3]	16 (14.8%)
Grade 2 [3 + 4]	80 (74.1%)
Grade ≥3 [≥4 + 3]	12 (11.1%)
Pretreatment risk stratification	
Low (low and intermediate)	64 (59.3%)
High (high and very high)	44 (40.7%)
Prostatectomy applied	
Open	45 (41.7%)
Laparoscopic	63 (58.3%)

**Table 2:** PSA response to radical prostatectomy in low- and high-risk patient groups. HT: half-life time of serum PSA in postoperative period. * PSA values measured below 0.002 ng/mL (low instrument sensitivity limit) were considered as 0.0 ng/mL.

Low Risk			
	PreOp	One Month	Three Months
PSA, ng/mL			
Mean	7.19 ± 5.038	0.04 ± 0.031	0.01 ± 0.011
Range	3.03–36.00	0.0 *–0.14	0.0 *–0.055
Median	5.80	0.033	0.003
HT, days			
Mean	n.a.	4.06 ± 0.661	34.26 ± 65.143
Range	n.a.	2.79–6.62	−282.67–282.75
Median	n.a.	4.02	31.32
**High Risk**			
	**PreOp**	**One Month**	**Three Months**
PSA, ng/mL			
Mean	10.84 ± 12.886	0.23 ± 0.858	0.27 ± 1.015
Range	2.15–67.71	0.0 *−5.58	0.0 *−6.32
Median	7.4	0.044	0.018
HT, days			
Mean	n.a.	5.24 ± 4.933	−35.34 ± 329.773
Range	n.a.	2.17–35.53	−1706.13–606.10
Median	n.a.	4.17	28.77

**Table 3:** Comparison of immunophenotype response to radical prostatectomy in low- and high-risk patient groups.

Low Risk				
Subset	PreOp (Cells/µL) Median (Range)	Three Months (Cells/µL) Median (Range)	*p* Value	Trend
CD3+	1272 (930–1649)	1433 (1139–2000)	<0.001	↑
CD4+	779 (573–1207)	887 (710–1228)	0.15	≈
CD8+	503 (356–711)	692 (449–920)	<0.05	↑
Ratio CD4+/CD8+	1.8 (1.1–2.4)	1.5 (0.9–2.3)	<0.05	↓
CD8+CD69+	185 (114–305)	196 (152–278)	<0.001	↑
Ratio CD4+/CD8+CD69+	4.5 (2.9–6.9)	4.3 (3.0–7.0)	0.33	≈
CD4+CD25+FoxP3+	3.1 (1.6–5.6)	3.3 (1.8–5.5)	0.76	≈
CD3-CD16+CD56+	232 (113–360)	317 (176–484)	<0.05	↑
MDSC	11.9 (4.7–21.4)	12.1 (6.0–21.7)	0.12	≈
CD19+	206 (119–337)	189 (110–285)	0.07	≈
**High Risk**				
**Subset**	**PreOp (Cells/µL)** **Median (Range)**	**Three Months (Cells/µL)** **Median (Range)**	***p* Value**	**Trend**
CD3+	1426 (967–2161)	1388 (1043–1852)	0.11	≈
CD4+	894 (587–1425)	803 (683–1074)	0.42	≈
CD8+	571 (342–925)	594 (405–920)	0.24	≈
Ratio CD4+/CD8+	1.6 (1.2–2.4)	1.7 (1.0–2.1)	0.81	≈
CD8+CD69+	205 (120–373)	227 (156–308)	<0.001	↑
Ratio CD4+/CD8+CD69+	4.3 (3.0–7.1)	3.9 (2.8–6.4)	0.07	≈
CD4+CD25+FoxP3+	4.6 (2.1–7.3)	3.9 (2.4–7.1)	0.11	≈
CD3-CD16+CD56+	211 (119–320)	328 (225–458)	0.14	≈
MDSC	12.4 (6.5–19.9)	16.2 (10.5–26.1)	<0.002	↑
CD19+	188 (124–299)	181 (147–272)	0.50	≈

Symbols in Table 3: “≈”—no significant change; “↑”—increase; “↓”—decrease.

**Table 4:** Comparison of immunophenotype responses to radical prostatectomy performed by the open or laparoscopic technique.

Open Radical Prostatectomy				
Subset	PreOp (Cells/µL) Median (Range)	Three Months (Cells/µL) Median (Range)	*p* Value	Trend
CD3+	1280 (878–2086)	1538 (924–1956)	0.84	≈
CD4+	826 (549–1400)	839 (630–1162)	0.72	≈
CD8+	554 (298–928)	711 (423–958)	0.15	≈
Ratio CD4+/CD8+	1.7 (1.2–2.4)	1.5 (0.8–2.0)	0.07	≈
CD8+CD69+	169 (92–347)	198 (159–261)	0.26	≈
Ratio CD4+/CD8+CD69+	4.5 (2.9–6.9)	4.3 (3.0–7.0)	0.16	≈
CD4+CD25+FoxP3+	3.4 (2.1–6.8)	3.5 (2.1–4.8)	0.30.	≈
CD3-CD16+CD56+	205 (106–329)	294 (170–441)	0.03	↑
MDSC	11.1 (4.7–16.0)	14.8 (10.5–26.1)	<0.01	↑
CD19+	188 (124–299)	180.735 (147–272)	0.74	≈
**Laparoscopic Radical Prostatectomy**				
**Subset**	**PreOp (Cells/µL)** **Median (Range)**	**Three Months (Cells/µL) Median (Range)**	***p* Value**	**Trend**
CD3+	1292 (964−1867)	1346 (1126–1984)	<0.05	↑
CD4+	780 (651–1282)	881 (696–1187)	0.46	≈
CD8+	538 (379–780)	576 (437–904)	<0.001.	↑
Ratio CD4+/CD8+	1.8 (1.1–2.4)	1.7 (0.9–2.3)	0.10	≈
CD8+CD69+	209 (131–330)	216 (152–322)	0.15	≈
Ratio CD4+/CD8+CD69+	4.0 (2.7–6.1)	4.2 (3.0–6.3)	0.10	≈
CD4+CD25+FoxP3+	2.37 (1.1–4.6)	2.5 (1.2–4.1)	0.91	≈
CD3-CD16+CD56+	223 (117–353)	359 (199–502)	<0.001	↑
MDSC	11.0 (6.4–14.9)	11.2 (7.4–21.7)	0.16	≈
CD19+	187 (119–307)	185 (131–273)	0.50	≈

Symbols in Table 4: “≈”—no significant change; “↑”—increase.

## Data Availability

Data supporting reported results can be found on: https://home.mycloud.com/action/share/b8cb1db3-d4c1-49c3-800a-97a4eb48a411.
